# Effect of Anode Material on Electrochemical Oxidation of Low Molecular Weight Alcohols—A Review

**DOI:** 10.3390/molecules26082144

**Published:** 2021-04-09

**Authors:** Marta Wala, Wojciech Simka

**Affiliations:** Faculty of Chemistry, Silesian University of Technology, B. Krzywoustego Str. 6, 44-100 Gliwice, Poland; marta.wala@polsl.pl

**Keywords:** electrooxidation, methanol, ethanol, propanol, ethylene glycol, fuel cell

## Abstract

The growing climate crisis inspires one of the greatest challenges of the 21st century—developing novel power sources. One of the concepts that offer clean, non-fossil electricity production is fuel cells, especially when the role of fuel is played by simple organic molecules, such as low molecular weight alcohols. The greatest drawback of this technology is the lack of electrocatalytic materials that would enhance reaction kinetics and good stability under process conditions. Currently, electrodes for direct alcohol fuel cells (DAFCs) are mainly based on platinum, which not only provides a poor reaction rate but also readily deactivates because of poisoning by reaction products. Because of these disadvantages, many researchers have focused on developing novel electrode materials with electrocatalytic properties towards the oxidation of simple alcohols, such as methanol, ethanol, ethylene glycol or propanol. This paper presents the development of electrode materials and addresses future challenges that still need to be overcome before direct alcohol fuel cells can be commercialized.

## 1. Introduction

With the growing world population and technological development, energy demands are constantly increasing; therefore, developing more sustainable energy sources is one of the greatest technical challenges of the 21st century. Conventional solutions are becoming increasingly suboptimal because of their high environmental impact, which strongly affects climate and leads to the acceleration of climate change

For these reasons, research on new energy sources is necessary. In particular, solutions that would enable the usage of streams that are now considered waste, like simple organic compounds, such as urea, methanol or ethanol, as fuels would be very beneficial.

The use of low molecular weight alcohols as energy sources has many advantages: they are liquids, which simplifies their storage and transport. They have high energy densities, which means that small amounts of these compounds provide large quantities of energy compared to conventional fuels.

The use of lightweight alcohols as a fuel goes back to the 19th century, as it was the fuel recommended by Otto when he has developed the spark-ignition engine [1]. In such an engine, methanol has lower air consumption (14.55 kg/kg vs. 6.5 kg/kg) and a higher octane number (97.7 vs. 108.7) than conventional gasoline [2]. For more advanced engine technologies, such as internal combustion engines, methanol and ethanol have been considered as fuel since their invention and have played the role of the fuel blends that have increased the octane number when added to the gasoline [3]. Both methanol and ethanol have been proposed as blendstocks for diesel fuel and gasoline [1]. Both have been used for biodiesel production [1].

Technologies for the large-scale production of lightweight alcohols have been known for centuries and are well developed [4]. Additionally, they are present in waste streams of many large-scale industrial processes, such as wood pulping [5].

**Figure 1:**
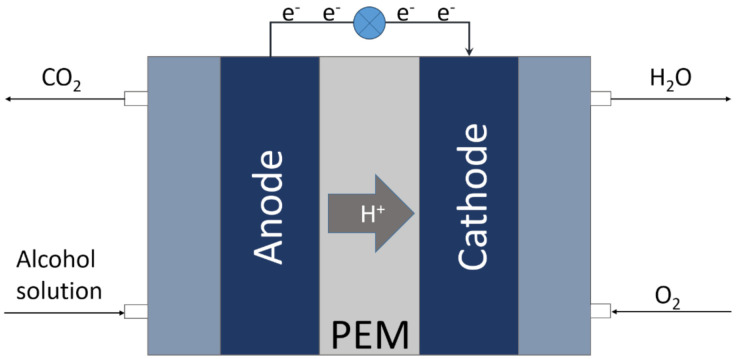
Scheme of a direct alcohol fuel cell.

All reasons mentioned above lead to the conclusion that simple organic compounds, such as low molecular weight alcohols, could possibly play the role of one of the main fuels in the future, but the usage of modern fuels requires modern solutions of power generation. The simple burning of these compounds for conventional power generation could lead to large energy losses., so gathering energy directly from a controlled reaction would be much more beneficial. This idea is the foundational concept of fuel cells (Figure 1). Fuel cells are devices that allow electrons to be directly gathered from oxidation reaction, which is taking place on its anodic pole [6].

In working direct alcohol fuel cell, the alcohol solution is fed on the anodic site of the cell and air or pure oxygen that is fed on the cathodic site. As the alcohol oxidizes on the anode, obtained electrons are moving through the outer electric circuit to the cathode, and protons are transported through the proton exchange membrane (PEM) to the cathodic part of the cell. There they react with hydroxide ions, which are the product of cathodic oxygen reduction. Water, which is a product of such reaction, is removed from the system with the cathodic gas stream [7,8,9].

Unfortunately, fuel cell technology still needs some improvement before it can be used as a large-scale solution. The main problem is related to the lack of electrode materials, mainly for anodes, which should be not only durable in reaction conditions and inexpensive but also show catalytic properties towards fuel oxidation. The development of such materials is a very complicated and time-consuming process that requires extensive research. A general state of anodic materials for fuel cells, as well as further specific information about materials for the oxidation of the selected compounds, is presented in this paper. They are divided into three main sections:

1. Structure of anodic material that describes the influence of morphology and structure of anodic materials on its properties;
2. Electrooxidation of alcohols is further divided into four subsections—methanol, ethanol, ethylene glycol and propanols. They contain a description of the sources of each fuel, mechanism of its oxidation, most popular materials used for its oxidation and direction of development necessary for the commercialization of fuel cells based on each alcohol;
3. Comparison of discussed alcohols that shows advantages and disadvantages of each alcohol as a fuel.

## 2. Structure of Anodic Material

Not only the electrode composition but also its structure strongly affects its catalytic activity. Material properties, such as the metal content, interatomic distance, band vacancy and number of neighboring metal atoms, are responsible for the chemical and electrochemical reactivity of electrodes [10]. One of the methods to enhance the overall electrode reactivity is doping. The addition of other metallic elements and the creation of bi- or trimetallic systems changes the crucial electrode properties, such as conductivity or surface activity [11,12,13,14]. Especially the electrocatalytic activity of noble metals can be enhanced by a synergistic effect between noble and transition metals thanks to the electronic (ligand) effect [15]., which happens in multimetallic systems because of different electronegativity of their components [16]. Such difference results in charge transfer from less electronegative transition metals to unoccupied valence orbitals of more electronegative noble metals, which changes the properties, such as adsorption strength, of the final material [16]. Easier desorption leads to higher catalytic activity because more active centers are accessible for the reaction. Additionally, alloys consisting of these metals show and show different intermetallic surfaces than pure, noble metals [16,17,18,19,20,21]. The properties of alloys can be designed by coupling their ingredients in the right proportions, which provides wide opportunities for tailoring alloy functions and enhancing their application performance [10,11,12]. Moreover, some metals can be present on the electrode surface in the form of oxides, preventing the poisoning by carbon monoxide that can occur during the oxidation of organic compounds [10].

**Figure 2:**
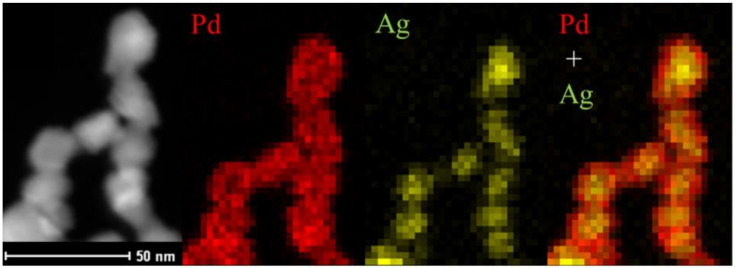
Compositional mapping images of PdAg@Pd core–shell structures, reprinted with permission of Elsevier [26].

Doping is not the only way to improve the performance of catalytic materials. Other properties that can be easily influenced and strongly change by the electrooxidation process include the properties of the electrode–electrolyte interface and the active surface of the catalytic material. The electrode surface area (ESA) can be influenced by changing the size of the catalytic particles. One of the most popular methods for increasing the ESA is the usage of catalytic material in the form of nanoparticles because of their high surface-to-volume ratio, which results in higher activity and immunity to poisoning in the final material [22,23,24]. There are almost endless possibilities regarding the shapes of nanomaterials, but the most popular ones are one-dimensional nanomaterials (nanowires) [25,26], nanocubes [24,27], nanocrystals [23,28,29,30,31], spheres [23,32] and hexagons. Core–shell materials, like core–shell nanorods presented in Figure 2, are a special part of nanomaterials because their specific structure strongly changes the reactivity of the final material.

They consist of a core material that does not have direct contact with the batch solution and shell material that is responsible for the reactivity of such molecules. Because catalysis is a surface process, the presence of catalytically active material is crucial only on the surface of the material [26,28]. Core–shell materials take advantage of this property—cheaper metal is usually used as a core material, so the cost of the whole system can be lowered by reducing the usage of the expensive, active metal [26,28,32,33]. Additionally, nanocages, because of their porous walls and characteristic hollow structure, allow maximum efficiency in surface atom utilization, and the control of the surface structures can optimize the active catalyst centers [24].

The catalytic activity of anodic materials also strongly depends on the size of the particles and the morphology of the obtained surface, which is correlated to the number of active centers where the reaction takes place [32,34,35]. Because of the high surface-to-volume ratio, smaller particles provide a greater quantity of reaction centers without affecting the macroscopic dimensions of the electrode and also improve the use ratio of noble metals [12]. For this reason, electrodes built from immobilized nanocompounds have recently attracted extensive attention from researchers.

The support materials on which nanocompounds are also immobilized strongly affect their reactivity by changing their properties, like electroactive area or electron transfer, and in consequence, the overall performance of the final electrode material [34,36,37,38]. Highly porous support materials provide better conditions for reagents diffusion, ensure higher dispersion of catalyst and prevent agglomeration of embedded nanoparticles [34]. Enhanced nanoparticle dispersion leads to a higher electroactive area of the system because of a higher amount of active reaction centers available for the reagents [34,36]. The high electrical conductivity of the support material enhances the electron transfer through the electrode, which enhances the reaction kinetics and prevent nanoparticles oxidation [34]. Desired properties of the support material are good electrical conductivity, a large surface area, and high corrosion resistance, strongly interact with the catalyst material and facilitate simple catalyst regeneration [34].

The support materials can also interact with the catalyst nanoparticles leading to a synergetic effect that takes place when the effect of using two different catalyst materials together is higher than the sum of their usage as monometallic materials [37,39,40,41], and electronic effect, which happens in multimetallic systems because of different electronegativity of their components [17,18,19,20,21,37]. As a result of such interaction, catalyst activity differs because the changed electronic structure of the active sites changes the strength of the reagents adsorption, which strongly influences the reaction kinetics [17,18,19,20,21,42].

The support materials for nanoparticle immobilization can be divided into two main groups: carbon and noncarbon materials. Over decades, carbon materials have been used as electrode materials in low-temperature fuel cells because of their extraordinary physical properties, such as high surface area, low weight, chemical inertia and good conductivity, but the main disadvantage of carbon materials is their sensitivity to corrosion caused by electrochemical oxidation [12,38]. Noncarbon templates, such as mesoporous silica [43], metal oxides [34,44], nitrides [20,38] and phosphides [45,46], show better corrosion resistance and high melting points but are characterized by lower electrical conductivity. In addition, this group of support materials has a wide range of other advantages that carbon materials do not have.

For example, titanium-based support materials lead to higher CO tolerance than carbon support catalysts, but they are not used as often as carbon materials because their high molecular weight lowers the mass activity of the catalyst [37,38].

Noncarbon templates are being used when their advantages are more significant than the disadvantages of their presence [37,38,47,48]. A good example of this kind of material is titanium meshes, which show good conductivity and are electrochemically stable and, therefore, can be good support for a wide range of electrocatalysts. Mesh-based anodes consist of only one layer, which allows the electrode to be thin. Additionally, electrodes based on meshes do not need to contain Teflon because they are more hydrophilic than conventional electrodes [49].

Due to their low-cost, stable physical properties, large surface areas and good conductivity, carbon materials, such as reduced graphene oxide (rGO) [13,50,51], graphene nanosheets (GNS) [13], carbon nanotubes (CNTs) [13,50], multiwalled carbon nanotubes (MWCNTs) [13,52,53,54], functionalized mesoporous carbon [13], exfoliated graphite (EG) [55,56], pyrolytic graphite [54] and glassy carbon (GC) [57,58,59] are widely used as catalyst support materials for the electrooxidation of low molecular weight organic compounds, such as methanol, ethanol and propanol.

Reduced graphene oxide has a high surface area and good conductive properties and is characterized by the presence of oxygen-containing functionalities that help effectively disperse catalyst particles and reduces the risk of CO poisoning of the final material because of its hydrophilic nature [13,50,51].

Another disadvantage of carbon support materials is the fact that some of them need to undergo some pretreatments to enhance their properties; for example, better results in terms of nanoparticle size, adhesion and distribution have been noted for MWCNTs doped with nitrogen by its treatment with nitric acid [36]. Such operation not only provides surface functional groups, such as OH^−^ and COOH^−^, but also removes most of the impurities; however, it may lead to surface defects that worsen the corrosion resistance and electrical conductivity [36].

Without such treatment, despite its advantages, such as good current conductivity, good thermal and chemical stability, large surface area, strong mechanical properties and excellent corrosion resistance, the number of active centers on the MWCNT surface is too low to allow good nanoparticle dispersion [36]. For example, some trials for the different modification of MWCNTs with polydopamine were recently conducted. It was demonstrated that such operations enhance nanoparticle distribution and prevent their deposition as large conglomerates, which leads to higher active areas because more reaction centers can be modified with polydopamine, enhancing the particle distribution [36,60,61].

Another support material that has been demonstrated to work as a support material for alcohol oxidation catalyst are carbon nanocages (CNCs). Instead of classic carbon support, their usage as a support material has led to an almost two-fold higher peak current density from methanol oxidation. They can be easily synthesized by pyrolysis of polypyrrole with the usage of the MgO template [62].

The metal-free semiconductor g-C_3_N_4_—polymeric graphitic carbon nitride—is also a popular support material because of its low cost, simple preparation and optimal elemental composition of both carbon and nitrogen. These materials are characterized by versatile physicochemical properties and significant electrocatalytic properties, but because of the presence of numerous irregular holes in their structure, they have lower thermal and electrical conductivities than other carbon materials [63,64].

To use advantages of both metallic and carbon support materials, some composite support materials consisting of inorganic compounds mixed with carbon materials, such as TiCN–GO, have also been examined [38]. The obtained hybrid material not only has a larger surface area but is also more stable than TiCN and provides interconnected pathways during the electrode process due to graphene oxide. The material has a one-dimensional anisotropic morphology, which can enhance electron transport properties for the supported catalyst materials and enhance the mass transport properties of electrode structures. The inorganic part provides a barrier that protects carbon material from oxidation during fuel cell operation. This material also offers great Pt-support reactions, including MOR activity, along with improved CO poisoning immunity [38].

## 3. Electrooxidation of Alcohols

### 3.1. Methanol Oxidation

Methanol was one of the first organic compounds produced on a large scale. Since the 19th century, it has been obtained by destructive wood distillation, which is why it is commonly called “wood alcohol”. Later, in the 20th-century, synthetic methanol was produced on a commercial scale [1,2,65]. Currently, methanol is obtained mainly by catalytic synthesis from syngas with a yearly production scale of approximately 85 million tons [1,4]. Methanol is a very important raw material for the chemical industry—a large amount of the methanol produced is used as a solvent or substrate for synthesis reactions for chemicals, such as formaldehyde, methyl tert-butyl ether and dimethyl ether [2]. Additionally, technologies for the catalytic hydrogenation of carbon dioxide extracted from industrial exhaust gases to methanol are known, but even though substantial progress has been made in recent years, these technologies are still not used commercially [1,66,67,68,69]. Due to its high energy density (see Table 1), the energy industry uses methanol directly as a fuel, as a fuel additive or as a substrate in biodiesel production [1,70]. Methanol as fuel gained the world’s attention during the Arab oil embargo in 1973 [2,65]. Since then, methanol has been widely used as a fuel additive in diesel engines [1,70,71], biodiesel production [71] and as a fuel alternative in internal combustion engines [1,71]. Because it can be economically produced from the fermentation of biomass or agricultural waste, it is considered to be a renewable fuel [72,73].

Recently, electrochemical methanol oxidation reactions (MOR) have gained researchers’ attention because they can be used as a direct source of electricity with direct methanol fuel cells (DMFCs). In DMFCs, an electrolyte containing a methanol mixture is directly fed to the anode, on which the oxidation process can take place at atmospheric pressure and temperatures lower than 100 °C [38,74]. Due to the electrocatalytic properties of anodic materials, electric current related to the chemical reaction can be obtained directly without the use of additional devices, such as vapor turbines [34,38,74,75,76]. DMFCs are considered a promising energy source because of their low pollution emissions, simple operation and high efficiency of energy conversion [34,54]. Additionally, their unique properties, such as simple configuration, suitability for small and portable applications (e.g., electronics) and higher energy density than the best rechargeable batteries based on lithium compounds, suggest developing DMFCs as a potential replacement for lithium-based batteries [34,77]. Presently, catalysts based on PtRu are widely used for MORs, but slow reaction kinetics still inhibit developing this technology [34,38,77,78]. Unfortunately, this is only one of the problems that DMFC technology must face, and most of them are related to the anode material on which the reaction takes place. One of the most undesired phenomena that can occur in DMFC is carbon monoxide poisoning, which occurs when molecules of CO formed during methanol oxidation irreversibly adsorb on active anode sites [6,34,75,77,79,80,81]. Platinum-based electrodes are very prone to this phenomenon [34,80,81]. As a result, fewer active sites are available for bulk methanol molecules, leading to a decrease in electrode activity and, consequently, a lower reaction yield [6,16,34,35,75,77,79,80,81,82,83]. For these reasons, developing new anodic materials with electrocatalytic properties towards methanol oxidation is necessary, and researchers are still working on this subject to enable the commercialization of DMFCs.

Different reaction mechanisms may lead to different products, but during electrochemical methanol oxidation, different product compositions have been observed for the same reaction conditions. This suggests that reactions can take place through different pathways, such as [81,93,94]:

1. Stepwise dehydrogenation to adsorbed CO and subsequent oxidation to CO_2_;
2. Reaction along parallel, “direct” paths to CO_2_;
3. Partial oxidation to formic acid and/or formaldehyde.

**Figure 3:**
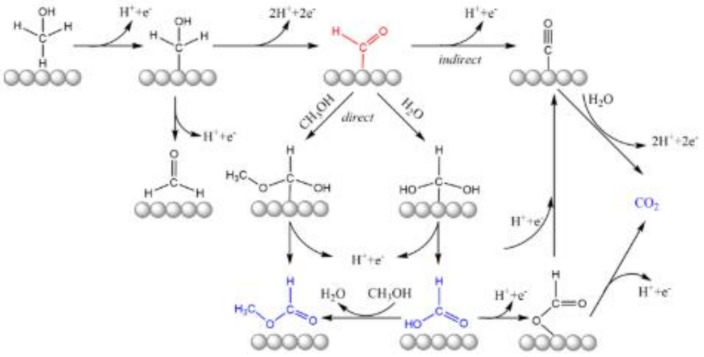
Proposed mechanism of methanol oxidation via direct and indirect way. Reprinted with permission of American Chemical Society [94].

All are presented in Figure 3. Regardless of the reaction path, the conversion rate towards carbon dioxide ranges from 90 to 100% [95].

Methanol oxidation can take place in both acidic and alkaline environments, but the mechanism of the reaction varies depending on the pH of the feed media. Since the anodic material should be immune to corrosion, during the process and without polarization, with a change in the supporting electrolyte, different anodic materials must be used. Below, different electrocatalytic materials are described with partitioning based on the pH of the supporting electrolyte.

In acidic solution, most of the catalytic materials are platinum-based. Because of platinum nobility, they remain stable in reaction conditions, but high-affinity of carbon monoxide derivatives to the platinum surface can lead to catalyst poisoning by permanent adhesion of oxidation products with electrode [19,21,96,97,98,99]. To prevent such a situation, different modifications of platinum electrodes have been proposed. Usually, good results are observed for bimetallic systems, where the addition of second metal leads to synergic [100,101,102], electronic (ligand) [59,78,103,104] or geometric effect [19,21] that changes the strength of adsorption of reaction products to platinum sites and thus prevent the catalyst poisoning or thanks to bifunctional mechanism increase the amount of adsorbed hydroxide ions, which enhances the further oxidation of CO_ads_ and thus prevent catalyst poisoning [21,99]. Added metals that will be mentioned later in the text usually have a higher affinity to oxygen than platinum and thus enhance the MOR and prevent catalyst poisoning.

The bifunctional mechanism of methanol oxidation on Pt-based catalysts can be explained by the example of a PtRu catalyst in acidic solution [7,34,62,78,94,105]:

Pt + CH_3_OH → Pt(CO)_ads_ + 4 H^+^ + 4 e^−^ (1)

Ru + H_2_O → Ru(OH)_ads_ + H^+^ + e^−^ (2)

Pt(CO)_ads_ + Ru(OH)_ads_ →Pt + Ru + CO_2_ + H^+^ + e^−^ (3)

CH_3_OH + 8 OH^−^ → CO_3_^2−^ + 6 H_2_O + 6 e^−^ (4)

In alkaline media, platinum also can play the role of anodic material, but because high pH is much less corrosive towards other metallic material, a non-noble catalyst, such as nickel, can be used [14,41,42,106,107]. Elimination of platinum not only reduces the cost of the catalytic material but also prevents catalyst poisoning with CO_ads_ since used metals have a lower affinity to carbon monoxide derivatives than Pt [41,42,106,107].

In alkaline media, both carbonate ions, as shown in reaction (4), and formate ions (reaction (5)) has been detected as a product of methanol oxidation [83]:

CH_3_OH + 5 OH^−^ → HCOO^−^ + 4 H_2_O + 4 e^−^ (5)

Reaction 4 takes place in a few steps with adsorbed species [83]:

CH_3_OH + 4 OH^−^ → (CO)_ads_ + 4 H_2_O + 4 e^−^ (6)

(CO)_ads_ + 2 (OH)_ads_ → CO_2_ + H_2_O (7)

and adsorbed hydroxide is present on the electrode surface as a result of a reaction (7) [83]:

OH^−^ → (OH)_ads_ + e^−^ (8)

As two adsorbed OH species are required for oxidization, one adsorbed CO molecule exchanges electrons to sum up to 6, which agrees with reaction (4).

Carbonate ions are present in a solution as a result of the following reaction [34,83]:

CO_2_ + OH^−^ → CO_3_^2−^ + H^+^ (9)

This reaction is particularly hazardous because the released carbonate ions can competitively adsorb on the electrode surface instead of methanol molecules and hydroxide ions. Additionally, the released CO_3_^2−^ leads to a decrease in the medium pH, which causes more sluggish reaction kinetics. For these reasons, alkaline DMFCs, despite their successful operation in space programs, have not been widely used on an industrial scale on Earth [83].

Complete methanol electrooxidation is a 6-electron pathway, reaction (4), with different steps and intermediate species, such as carbon oxide (reactions (1) and (5)), formaldehyde and formic acid (reaction (5)) depending on the process conditions. The formation of these intermediates causes slow reaction kinetics and low efficiency because the formation of every final product that is different from CO_2_ lowers the number of exchanged electrons (reaction (4) vs. reaction (5)), leading to a lower efficiency for the whole process [38,83].

The reactions shown above represent a so-called bifunctional mechanism that assumes that methanol adsorbs only on the surface of platinum, while on the surface of ruthenium, only water splitting takes place. This theory simplifies some phenomena, such as water oxidation on platinum in a higher potential range [96] and the adsorption of methanol on ruthenium at a higher temperature [96,108]. Additionally, adsorbed species can move on the surface and occupy Ru atoms even though they are primarily formed on the Pt surface [96]. This simplification is possible because the best results are observed if the final step of MOR takes place exactly, as shown in reaction (3)–between carbon monoxide adsorbed on the surface of platinum and hydroxide adsorbed on the surface of ruthenium. For this reason, the diffusion rate of (CO)_ads_ on the catalyst surface limits this reaction because this species must migrate towards the adsorbed hydroxide for the reaction to take place [96]. Commercially available catalysts are made of Pt and Ru in an atomic ratio of 1:1, but the proposed methanol oxidation mechanism suggests that three free platinum sites are needed for methanol adsorption and that one ruthenium free site is necessary for water splitting (reaction (2)), so the best results should be observed for catalysts containing platinum and ruthenium in atomic ratios from 3:1 to 1:1 [108]. Methanol oxidation on Pt–Ru reaches a maximum peak current for ruthenium contents between 15 and 45%. A further increase in Ru content leads to a decrease in the reaction current because too few platinum reaction centers are available for methanol molecules to adsorb [105].

The oxidation of the adsorbed carbon monoxide derivative reaction (7) is the limiting step for the MOR, especially on platinum-based catalysts [83,109]. This reaction strongly depends on the amount of adsorbed OH species because if most of the reaction sites are taken by hydroxide species, too few free reaction sites are available for methanol particles; however, if too few hydroxide anions are adsorbed, the reaction cannot be optimized [83].

The potential at which methanol oxidation takes place can strongly influence the contribution of each possible product. For the Pt (111) catalyst in alkaline media, at 0.4 V, formate is observed to be the main product, but at approximately 0.5 V, carbonate is detected as the dominant product. Additionally, in the low potential range, the amount of adsorbed OH species is so high that the reaction rate decreases as a result of oxidation of adsorbed OH ions into electrochemically inactive oxide [83].

Because MOR is very sensitive to the catalyst surface structure [96] for platinum catalysts with structures other than (111), potential values of approximately 0.6 V have led mainly to the formation of formate [83]. This difference is related to the varying coverage of the platinum surface with CO species depending on the catalyst structure. The slowest methanol dehydrogenation (reaction (6)) takes place on a Pt(111) surface, which makes it the least covered with CO species among basal structures—Pt(100) and Pt(110). This phenomenon is related to the number of defects on the platinum surface since defects are the active sites for methanol dehydrogenation. Different types of defects promote different reactions; for example, kink-type defects on Pt(111) structures promote CO oxidation, and step-type defects on the same structure promote methanol dehydrogenation. This means that by controlling the surface structure, we can control the CO coverage of the electrode surface and, by that, the reaction path and its rate [81,83,96].

Additionally, at higher temperatures and methanol concentrations, dimethoxymethane and methyl formate have been identified as products of methanol and formaldehyde reactions. The current efficiency and ratio of different intermediates strongly depend on the reaction conditions and type of electrode or catalyst [96].

Platinum- and platinum-based nanomaterials have been widely used as anode materials for methanol oxidation in DMFCs [38,54,83]. In acidic media, the electroactivity is the highest, but at the low temperatures at which DMFCs function, carbon monoxide poisoning readily occurs.

To overcome this disadvantage, Pt-based electrodes have been improved with the addition of p- or d-orbital elements such as Ru [21,96,97,105], Pd [74], Ni [21,99], Fe [42,66], Cu [19,94], B [77], Au [64], Nb [78] and Co [12,21,54,62] Mo [21,96] or Sn [21,97,105] as cocatalysts. Among the platinum-based alloys, Pt–M intermetallic compounds are attracting attention because of their high activity as fuel cell anodes, especially methanol [110,111,112,113]. The addition of these metallic elements lowers the onset MOR potential and boosts the peak current density, which leads to higher reaction yields and eventually higher DMFC efficiency. Additionally, additives can change the CO adsorption sites and thus prevent CO poisoning. Not only bimetallic but also ternary systems have been studied, and the presence of a third metallic element can significantly improve the kinetics of the MOR, i.e., Pt–Cu–Fe/C electrodes show lower CO adsorption and lower MOR onset potential than their disoriented counterparts and Pt/C catalysts [94]. Platinum properties can be changed not only by doping pure metals but also by the presence of their oxides. If metal oxides (i.e., RuO_2_, MnO_2_, MoO_2_, and IrO_2_) are used for platinum embedding, they alter the electrochemical features of the obtained electrode (electronic effect), which changes the adsorption energy of methanol and thus improve the kinetics of the MOR. This effect is caused by changing the conditions of proton and electron transfer by metal oxide hydration [34,112,114,115].

Because catalysis is a surface process, there is a chance of lowering the overall cost of catalysts using different materials underneath the active layer, for example, by using core–shell nanostructures. The core material should be resistant to the process conditions and cheaper than the shell material. Many core–shell materials have been studied; for example, Pt@Ru and PtRu@IrNi core–shell materials have been tested as methanol oxidation catalysts in acidic media. The proposed materials show better catalytic properties towards MOR than commercially available materials, even without the preferred surface composition (Pt:Ru 3:1) [108].

Not only the composition of the electrode surface but also its roughness affects the MOR. Smooth Pt electrodes show an enhanced yield of partial MOR leading to formic acid and formaldehyde. The same effect is observed for enhanced mass transport conditions and ambient temperatures. This also shows that laboratory results from experiments on smooth electrodes and under ideal conditions are poorly related to the reaction characteristics under DMFC operational conditions [93].

Although platinum-based catalysts yield the best results for MOR in acidic media, Pt shortages and high prices have compelled researchers to look for nonplatinum electrocatalytic materials. In addition, Pt-based electrodes may catalyze the formation of HCOOCH_3_, either by catalyzing the esterification reactions between formic acid and adsorbed methanol or as a result of the nucleophilic attack of CH_3_O^−^ on adsorbed HC = O [94]. Increasing the Pt content increases the HCOOCH conversion efficiency linearly [94]. For the mentioned reasons, attempts to obtain a nonplatinum electrode material for acidic media have been made. For example, Co–Pd/Sn/RGO active electrocatalysts obtained by electroless deposition of cobalt nanoparticles on RGO can be cost-effective nonnoble electrodes for methanol oxidation for DAFC with the use of acidic media [10].

Better results for the usage of nonnoble metals as anode materials for MOR have been reported for alkaline media [54]. Alkaline media not only enable better kinetics of methanol oxidation than acidic ones but also are coupled to weaker poisoning effects [54,83]. Even more important, alkaline media enable the usage of cheaper materials, such as nickel [116,117,118] or copper [119,120,121], as anodes because under these conditions and MOR leads to fewer intermediates than in acidic media. The alkaline environment is much less corrosive for nonnoble electrode materials, and thus, the overall process costs are lower [54,83]. Additionally, in alkaline media, anion adsorption is weaker, enhancing the main oxidation reaction because more free active sites on the surface of the electrode are accessible for methanol particles [83].

For alkali media, Pt can also be used as a catalyst material, but similar to acidic media; some additives have been used to enhance its catalytic activity under these conditions. Because of the alkaline environment, water splitting is no longer necessary for the presence of (OH)_ads_, which leads to the usage of different metals for doping Pt electrodes. In contrast to acidic media, the addition of Ru does not improve platinum catalyst properties towards MOR [83], but the presence of Ni significantly improves platinum catalyst performance [122]. Other metal dopants, such as gold [83] or silver [50], have been tested as platinum catalyst additives. In the case of gold, a synergic effect has been observed, leading to obtaining the same electrode activity of 75% Au and 25% Pt on carbon support as Pt/C has been reported [83].

Regardless of their excellent performance, platinum-based catalysts are too expensive when large-scale applications are considered. Additionally, if platinum shortages are taken into account, it becomes clear that other electrocatalytic materials for DMFC anodes must be found [34,108].

The most popular anodic materials for alkaline media are nickel [106,118,123,124,125,126] and cobalt-based electrodes [10,106,107,123,126,127], but other metals, such as gold [35,57], have also been studied. Similar to platinum, their properties can be changed by doping with other elements, both metallic, such as the Pd–Co [128] system and Ni–Cr_2_O_3_ [129], and nonmetallic, such as borides [123] and phosphates [106].

The slow kinetics of methanol electrooxidation lead to lower power densities of direct methanol fuel cells. Using anode materials that indirectly oxidize methanol (such as nickel or cobalt) accelerates MOR kinetics and results in a higher power density in fuel cells [130,131]:

NiOOH + CH_3_OH + 1.25 O_2_ → Ni(OH)_2_ + CO_2_ + 1.5 H_2_O (10)

CoOOH + CH_3_OH + 2.5 O_2_ → 2 Co(OH)_2_ + 2 CO_2_ + 3 H_2_O (11)

Usage of nickel and cobalt together shows interesting results [106,126,131,132,133,134]. This effect is a result of the reaction between these two hydroxides:

Ni(OH)_2_ + CoOOH → NiOOH + Co(OH)_2_ (12)

The presence of both of these metals in electrode material leads to an increased number of active sites for methanol oxidation on the electrode surface enhances the reaction kinetics [106,126,131,132,133,134].

Additionally, systems containing three active components have been tested, such as Pd-Cu-Co [128] and NiCoPO [106]. The addition of cobalt to the Ni–PO system lowers the onset potential even more, depending on the cobalt-nickel proportions [106].

### 3.2. Ethanol Oxidation

Despite mentioned advantages, direct methanol fuel cells still have some obstacles to overcome. The most important factors are the high overpotential of the methanol oxidation reaction (even with the usage of catalytic anode materials) [135,136], carbon monoxide catalyst poisoning for platinum-based materials [136] and the high methanol crossover rate, which impede cathodic performance [135,136]. To overcome these problems, new electrocatalytic materials should be prepared, or other low molecular weight alcohols can be used as liquid fuels for fuel cells

Among other liquid fuels for direct fuel cells, ethanol has the greatest chance for practical application. It has an even higher energy density than methanol (8,27 kWh/kg; see Table 1) and is a nontoxic liquid with high permeability [32,136]. The concept of using ethanol as a fuel has been known for years, and the idea of using agricultural alcohol as a fuel has been considered for a long time. For example, after the Bolshevik Revolution, this idea was strongly supported by leaders, but it met strong resistance from citizens, who did not want their beloved vodka to be “misused” [66]. In some countries, i.e., Brazil, ethanol is already used as a fuel for combustion engines. In these cases, the fast application of direct fuel cells as an energy source in vehicles would be simple because no changes in existing infrastructure would be necessary [83,135]. The production of ethanol is one of the oldest biochemical processes used on an industrial scale. It can easily be produced in large quantities by fermentation of any plant-based material [1,32,135]; these materials can be divided into two main feed streams: starch-based feedstocks (corn, grain, and barley) and sugar-based feedstocks (sugarcane and cane citrus molasses) [1]. Moreover, for industrial purposes, ethanol can be obtained by the direct and indirect hydration of ethylene with phosphoric or sulfuric acid as a catalyst [1].

Ethanol molecule consists of two carbon atoms that are connected by a strong inter-carbon bond. To fully oxidize this molecule, not only the bond between oxygen and hydrogen, as in the case of methanol, must be broken, but also this strong C–C bond. The durability of this bond is responsible for ethanol’s stability, which makes it a perfect fuel; however, at the same time, it is the main source of the challenges to using ethanol’s full potential as a current source [83,100].

The electrochemical ethanol oxidation reaction (EOR) is more problematic than the MOR because the strong bond between two carbon atoms must be destroyed in addition to the bond between hydrogen and oxygen.

Electrooxidation of ethanol occurs through different pathways and thus results in different products depending on the reaction regime—the electrode potentials, feed stream composition and temperature [135,137]. The complete oxidation of ethanol to carbon dioxide is a 12 electron reaction, so it should be twice as efficient in terms of current income as methanol oxidation. Complete oxidation, where carbon dioxide is the main carbon-based product, represents the so-called C1 mechanism and is the goal of DEFCs [135,137,138]. Complete oxidation allows the maximum usage of oxidized fuel by providing the highest number of electrons from one molecule of the fuel [7]:

CH_3_CH_2_OH + 3 H_2_O → CO_2_ + 12 H^+^ + 12 e^−^ (13)

Unfortunately, for ethanol oxidation, most catalytic materials are not selective enough to break the C–C bond, and instead, the C2 mechanism reaction takes place, where the main products are acetic acid (reaction (14)) and acetaldehyde (reaction (15)) that provide only 2 and 4 electrons, respectively [7,135,137,138,139]:

CH_3_CH_2_OH + 3 H_2_O → CH_3_COOH + 4 H^+^ + 4 e^−^ (14)

CH_3_CH_2_OH → CH_3_CHO + 2 H^+^ + 2 e^−^ (15)

**Figure 4:**
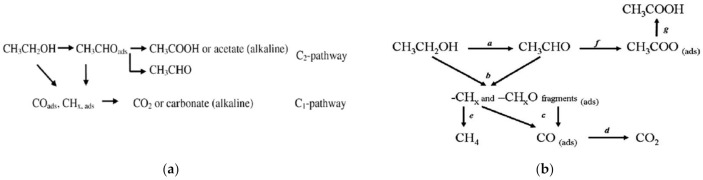
Mechanism of ethanol oxidation in (**a**) alkaline and (**b**) acidic media reprinted with permission of Elsevier [136].

The possible products of ethanol oxidation are carbon dioxide, acetaldehyde and acetic acid, but the carbon dioxide distribution is low than that of methanol oxidation (where it is approximately 90 to 100%) [135]. Mechanisms of ethanol oxidation in acidic and in the alkaline environment are shown in Figure 4.

Additionally, a subsequent reaction between ethanol and ethanal, which leads to the formation of diethyl acetal, is possible and results in lowering the process efficiency [135]:

CH_3_CHO + 2CH_3_CH_2_OH → CH_3_CH(OCH_2_CH_3_)_2_ + H_2_O (16)

The oxygen necessary for CO_2_ formation is provided by water molecules, which are strongly visible in reaction (13). Increasing the participation of water in the feed stream is beneficial only to the maximum, limiting value. For water-to-ethanol mole ratios higher than 5:1, the ethanol partial pressure can decrease, which can lead to mass transport problems [135]. The presence of water promotes more current efficient reactions (reaction (13) and (14)) of ethanol oxidation, and, in its presence, only trace amounts of diethyl acetal have been found in the product stream. According to the Le Chatelier–Braun rule, in the presence of water, the equilibrium of reaction (16) is strongly shifted to the left side; therefore, very little or no ethanol diethyl acetal is present in the product stream.

Temperature also has an impact on the reaction rate; at elevated temperatures (above 150 °C), reactions have been noted to be more current efficient [135].

From a fuel efficiency point of view, ethanol oxidation to carbon dioxide is the desired reaction in direct ethanol fuel cells, so research towards electrocatalytic materials for DEFCs anodes should focus on developing materials that catalyze the C1 reaction mechanism. However, the C2 mechanism of EOR, leading to obtaining acetaldehyde as a reaction product, can also be used in DEFC because such product would not accumulate in the environment since Mammals and yeasts are capable of producing enzymes that biologically degrade acetaldehyde to acetic acid and acetates. This amplifies the idea that acetaldehyde emissions from fuel cells will not cause acetaldehyde accumulation in the environment or in living species. However, acetaldehyde’s environmental impact would still be higher than the impact of carbon dioxide emitted from methanol-fed fuel cells [135].

Similar to the MOR, electrooxidation of ethanol can take place in the acidic and alkaline media. Anodic materials must be properly chosen and adapted to the reaction conditions. More researchers have focused on developing anodic materials for alkaline feed streams because in this kind of medium, with the chemical activity of ethanol, the oxidation rate is higher [32,136]. Additionally, a high pH inside the fuel cell is beneficial from a corrosion point of view because, in basic solutions, a wide range of anodic materials is more immune towards corrosion than in acidic ones [11].

Anodic materials for ethanol oxidation can be divided into two main groups: materials based on platinum [83] and materials in which palladium is the main ingredient [11].

Platinum, as an electrocatalytic material, provides a high number of active coordination sites and shows relatively high selectivity towards breaking the inter-carbon bonds of alcohols [138]. As mentioned, after adsorbing on the active sites of platinum, ethanol molecules can react through various pathways. They can either succumb to dissociation (C1 mechanism), which leads to strong adsorption of carbon monoxide derivatives (CO)_ads_ and CH_x_ intermediates on the surface of the electrode, or oxidation (C2 mechanism), which results in acetic acid and acetaldehyde. To further oxidize the adsorbed carbon species, the presence of adsorbed hydroxide ions is necessary. This complicated cascade of reactions is why ethanol oxidation is so problematic. The adsorption of ethanol and the further breakage of its C–C bonds are inhibited by the presence of adsorbed carbon species at low overpotentials and by (OH)_ads_ at high overpotentials, which is why the EOR regime should be strictly controlled by the electrode polarization potential and environmental composition [138].

Platinum-based nanocompounds are one of the most promising nanomaterials for electrocatalytic ethanol oxidation. Their main advantages are stability and predictable surface composition leading to predictable distribution of active centers. Unfortunately, Pt-based materials are very prone to carbon monoxide poisoning and lose their reactivity due to nanoparticle migration and agglomeration [138,140,141] and have a sluggish reaction rate. The doping of oxophilic elements, such as Sn or Ru, in platinum electrodes significantly improves their catalytic performance by enabling the adsorption of hydroxide ions at low overpotentials thanks to the bifunctional effect [138,140]. They also change the electronic structure of the electrode by decreasing the d-band center, which weakens the adsorption of CO intermediates [15,140], so they enable both bifunctional and electronic (ligand) effects [15,138,140]. Unfortunately, doping of this kind of element leads to the lowering of the catalytic selectivity towards the oxidation of ethanol to carbon dioxide [138].

Similar to PtRu in the case of methanol, platinum electrodes doped with tin are very popular materials for the electrooxidation of ethanol in acidic environments. Ruthenium-doped electrodes do not work in the case of complete ethanol oxidation because they are unable to break the C–C bond [16,136,139,142,143]. The incorporation of tin into a platinum catalyst changes the electrode geometric and electronic structure, providing conditions required for complete ethanol oxidation to carbon dioxide [15,16,143,144].

**Figure 5:**
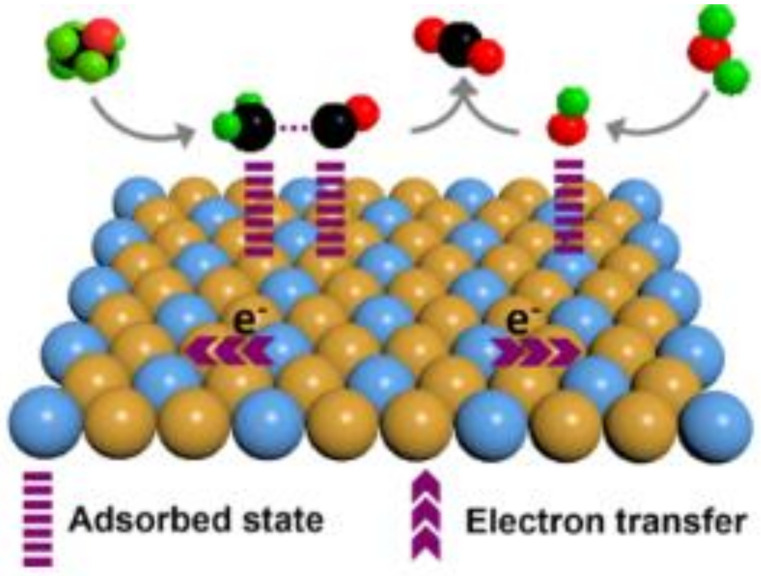
Bifunctional mechanism of ethanol oxidation reaction (EOR) on the PtSn (Pt—brown, Sn—blue) catalyst surface, reprinted with permission of American Chemical Society [15].

Due to natural differences in their electronegativity, charge transfer from less electronegative tin towards more electronegative platinum takes place. As a result, the unoccupied platinum 5d orbital is partially filled with 2d Sn electrons, and an electronic (ligand) effect takes place [15,16,144]. Modification of the unoccupied platinum d band leads to a lower affinity of carbon species towards platinum, which causes a decrease in catalyst poisoning by CO_ads_. Weaker bonding occurs not only between the Pt and carbon intermediates but also with all electroactive species; however, the decrease in platinum catalytic properties is balanced by tin catalytic properties and a weaker poisoning effect [15,16]. Additionally, CO can adsorb on the surface of Pt (111) in various forms, such as linear or bridged, but on the surface of Pt_3_Sn (111), because of the incorporation of tin atoms into the lattice, CO can adsorb only in linear form, which decreases the amount of CO adsorbed [15,16]. The presence of tin promotes the oxidation of alcohols by providing adsorbed OH species from water dissociation taking place at low potentials due to the presence of tin hydroxides [144]. Therefore, PtSn catalysts show a bifunctional oxidation mechanism, as shown in Figure 5, and an enhanced ability to break the C–C bonds in simple alcohols, such as ethanol [15,16,143,144]. The optimum tin content provides an optimal number of surface oxygen derivatives that are capable of oxidizing the adsorbed carbon intermediates and provides pertinent dilatation of the lattice parameter so that the C–C bond can be broken [16,144]. With increasing tin content, the current density obtained by ethanol oxidation increases, but only towards a specific maximum value. An Sn content that is too high leads to a decrease in active platinum sites or weakens the adsorption of alcohols on the Pt surface [16,136,144]. Unfortunately, the optimal ratio varies depending on the reaction temperature, potential range and catalyst type. The optimal tin content for ethanol oxidation in 0.5 M H_2_SO_4_ at room temperature reported by researchers varies from 10 to 50% depending on the electrode preparation procedure [15,16,143,144,145].

Tin in the form of SnO_2_ also enhances the catalytic properties of platinum electrodes. Generally, metal oxides mixed with Pt alter its electronic structure and enhance the removal of adsorbed carbon oxide intermediates from active platinum sites. One of the greatest advantages of tin and its compounds as doping agents is that the required processing is minimal [140].

Other forms of platinum–tin catalysts based on nanoparticles have also been developed. Ultrathin nanofibers of PtSn_3_ [15], PtSn nanospheres [143] or Pt–Sn nanostructured catalyst [16,144] have shown enhanced stability and activity towards ethanol electrooxidation. Especially platinum-based nanofibers are interesting from a fuel cell point of view because they are characterized by good structural foundations, such as flexibility, conductivity, large electroactive surfaces and inherent anisotropic morphologies. Additionally, this type of nanomaterial is less prone to aggregation or other structural deformation types that could cause serious damage during fuel cell operation [15].

In acidic media, some catalyst materials show even higher peak current densities for ethanol oxidation than for methanol oxidation, which is compliable with the theoretical assumption that ethanol can be a better current source than menthol. Pt-Sn catalysts show a tendency of increasing ability to oxidize alcohols as the tin content increases with increasing oxidized compound carbon atom number [16].

Among other oxophilic elements, nickel has shown interesting properties as a dopant in Pt electrodes. The obtained electrocatalytic material has higher activity than pure platinum, is less prone to CO poisoning and has a lower carbon dioxide to acetic acid ratio [138]. PtNi octahedrons are more specific towards the breaking of the C–C bond of ethanol and further oxidation towards carbon dioxide than pure platinum electrodes. This leads to a higher number of exchanged electrons, which results in an improvement in the overall process efficiency [138]. Similar properties have been observed for platinum electrodes doped with boron. These have better electrocatalytic properties and CO tolerance in acidic media than Pt [146].

One of the biggest problems of the catalysts based on bimetallic platinum alloys in an acidic environment is the leaching of the less noble metal from the alloy surface [147]. Such phenomenon can be prevented by the addition of another metallic element, such as gold [141].

Another way of preventing the CO poisoning of platinum electrodes is the usage of composite materials, such as Pt/r-NGO/NbN. Metals carbides and nitrates do not enhance the system CO toleration, but also provide high stability of the system, and because of their low cost, they do not increase the cost of the overall system [100]. Niobium nitride shows enhanced electrical conductivity and good stability in both acidic and alkaline media, which makes it a good doping agent. Because niobium in nitride molecules can go through three oxidation states—Nb (V), Nb (IV) and Nb (III)—Pt/r-NGO/NbN composites show strong electrooxidation properties. In comparison with commercial Pt/C catalyst Pt/rGO/Nb_4_N_3_ composites, Pt/r-NGO/NbN composites have higher peak current density, higher CO tolerance, lower onset potential and lower potential shift. Their enhanced immunity towards CO poisoning is probably related to a bifunctional mechanism in which water split takes place at the NbN sites, and the OH_ads_ produced in this reaction prevent the poisoning of the neighboring Pt sites [100].

Most platinum-based materials for ethanol oxidation in alkaline media lead to the C2 mechanism resulting in the formation of acetaldehyde and acetate as the main products, making them unprofitable for use as anodic materials for DEFCs [16,83,136]. They also show high sensitivity towards CO poisoning, which leads to a decreasing number of active sites available for ethanol and a decrease in the catalyst activity [16,83,136]. Additionally, platinum is a rare and strategic metal, and its price is relatively high, which makes Pt-based anodes expensive.

The commercialization of these electrodes can be difficult, and thus, more researchers have focused on developing Pd-based catalytic anodes for the EOR. Anodic materials based on palladium are an alternative to the widely used platinum-based anodes [11]. Palladium is a very promising metal regarding alcohol oxidation in alkaline solutions. It not only shows high catalytic activity and stability on its own but also, thanks to the synergic effect, enhances the performance of other metallic elements [32,136]. Because of these advantages, palladium bimetallic systems have been widely investigated because of their potential application as reaction catalysts and as anodic materials with electrocatalytic properties in fuel cells.

Ethanol oxidation over palladium to C2 products shows the following mechanism [12]:

Pd + OH^−^ → Pd-OH_ads_ (17)

Pd + CH_3_CH_2_OH → Pd-(CH_3_CH_2_OH)_ads_ (18)

Pd-(CH_3_CH_2_OH)_ads_ + 3 OH^−^ → Pd-(CH_3_CO)_ads_ + 3 H_2_O + 3 e^−^ (19)

Pd-(CH_3_CO)_ads_ + Pd-OH_ads_ → Pd-CH_3_COOH + Pd (20)

Pd-CH_3_COOH + OH^−^ → Pd + CH_3_COO^−^ + H_2_O (21)

The final product of this cascade of reactions is acetate, which is the main reason why palladium is not used as an EOR anodic material on its own. The addition of another metallic element to the Pd catalyst makes it more stable and more active towards ethanol oxidation in alkaline media because it increases the ability of the final material to break the C–C bond [136].

For C1 products, a reaction between adsorbed (CH_3_CO)_ads_ and OH_ads_ look differently [148]:

Pd-(CH_3_CO)_ads_ + Pd-OH_ads_ + 6 OH^−^→ 2 CO_2_ + 6 H_2_O + 4 Pd + 6 e^−^ (22)

Comparing reaction (20) to reaction (22), the importance of the hydroxide ions becomes clear. It not only provide conditions that enable ethanol oxidation towards C1 products but also allows usage of less expansive, non-noble metals as electrode materials [10].

Palladium-based catalysts can be used in acidic media. For example, Co-Pd/Sn anodes have been proven to have significant electrocatalytic activity for the EOR [10], but because of advantages that come with the usage of alkaline media that have been mentioned above, most of the researchers have focused on developing electrocatalytic materials for alkaline media [10].

Different metals have been investigated as palladium cocatalysts for ethanol electrooxidation in alkaline media.

Copper is an inexpensive metal showing catalytic properties towards ethanol oxidation, making it a promising cocatalyst for palladium [13,32,148,149]. The presence of copper in the catalyst is leading to the electronic (ligand) effect, which enhances the performance of the anodic material [13]. The use of core–shell nanoparticles, where a PdCu alloy mixed with pure palladium is the shell material, and copper is the core, has shifted the performance, even more, thanks to a higher active area related to a higher surface-to-volume ratio [32]. The addition of copper has not only increased the oxidation peak current density but also increased durability in alkaline media and the immunity towards CO poisoning [32].

Platinum also has been tested as a cocatalyst material for Pd-based anodic materials for ethanol oxidation because they combine the advantages of both materials—the high activity of both platinum and palladium towards alcohol oxidation and enhanced immunity towards CO poisoning thanks to the presence of Pd [11,137,150,151,152]. According to the literature, the rate-determining step in ethanol electrooxidation is the reaction between the adsorbed CH_3_CO_(ads)_ and adsorbed OH_(ads)_ (reaction (20)) [11,137] or breaking of the C–H bond to obtain C1 products [151]. The catalytic effect observed for the PdPt catalyst is probably the result of an electronic effect where the d-band palladium center is shifted in the presence of platinum. Shifted Pd centers promote OH adsorption, which increases the rate of adsorbed species reaction and thus improves the overall reaction kinetics [11].

Doping with silver leads to electrocatalytic materials that are more active and more stable towards the EOR in alkaline environments [26,136,153,154]. The addition of silver to a palladium catalyst leads to a lower potential of the reaction peak than pure Pd under the same conditions, which may contribute to the acceleration of the reaction rate and the smaller size of the obtained catalytic particles, leading to a larger electroactive surface on the working electrode [26,136,153,154]. To obtain a larger electroactive surface on such electrodes, different core–shell structures, such as PdAg@Pd core–shell worm-like structures, which are shown in Figure 2, have been developed [26]. Because of its structural properties, this type of nanomaterials has shown better stability and higher residual activity and better noble-metal utilization than other catalysts [26,136,153,154].

Nickel as a metal shows catalytic properties towards the oxidation of alcohols and other low molecular weight organic compounds [12,13,136,155,156,157,158]. When nickel is added to alkaline media at the reaction potential, it oxidizes into nickel hydroxide, which increases the surface coverage of OH_ads_ species, leading to an increasing overall reaction rate since the reaction between the adsorbed species on the palladium surface is the rate-determining step [12,157]. The addition of nickel not only increases the amount of adsorbed hydrogen ions but also changes the electronic structure of palladium thanks to the electronic (ligand) effect, which improves the catalyst reactivity and its immunity towards CO poisoning [12]. The dissociative adsorption of ethanol proceeds quickly, so the rate-determining step of this process is the reaction between the adsorbed hydroxyl groups and adsorbed acyl groups, which leads to the removal of the adsorbed species [12]. The use of PdNi nanoparticles has shifted the catalytic properties of the material thanks to a higher volume to surface ratio and allowed better usage of strategic and expensive noble metals [12,156]. Even better results were observed for PdNi materials doped with phosphorus because of their enhanced activity and stability related to the presence of phosphorus [13,155] that, as a nonmetallic element, is capable of modifying the metals electronic structure, which enhances the electronic (ligand) effect related to the presence of nickel in PdNi catalyst [13].

Nickel itself can be used as a catalytic material. Because of its low price (compared to platinum), it has been proposed as the first nonprecious metal for alcohol oxidation and is gaining increasing research attention. The nickel surface characteristics, related to its oxidation from Ni^2+^ to Ni^3+^, lead to strong results in regard to the oxidation of simple alcohols [134]. During alcohol oxidation, Ni^3+^ is the active species, so the performance of nickel-based electrodes can be further improved using another element with low oxidation potential as a dopant, which enhances the oxidation of nickel from the II to III oxidation state [134]. Examples of these elements include cobalt [134], chromium [129] and molybdenum [159]. In the case of a cobalt bifunctional mechanism takes place because Co atoms promote the adsorption of hydroxide ions at low potentials and thus improve the formation of nickel hydroxide active sites, as it was shown in reaction (10) [134].

Ni–Co–Fe has been used as an industrial-scale ethanol oxidation catalyst with the trade name HYPERMEC^TM^ [83,160]. The producer—an Italian company Enapter (previously called Acta) declares a peak performance of over 250 mW cm^−2^ at 80 °C with ethanol fuel and fuel cell durability over 3000 h for their noble metal-free catalyst [160]. They have also supplied electrodes for probably the world’s first fuel cell demonstration vehicle in cooperation with a team from the Hochschule Offenburg—the University of Applied Science in Germany at Shell Eco Marathon in France in 2006 [160]. The use of fuel cells as a power source for vehicles is very important because ethanol usage by the automotive industry is already increasing with the increasing participation of biodiesel, and further changes towards the elimination of fossil fuels would be much easier [83,160].

During its usage of HYPERMEC (Ni–Fe–Co catalyst), no acetate is found as a reaction product, and the formed acetaldehyde is further oxidized to carbon dioxide. Additionally, the lack of CO poisoning and higher current density obtained during the EOR with this system are large advantages compared to Pt-based catalysts [83]. The proposed reaction mechanism suggests that Ni sites are responsible for ethanol dehydrogenation and the breaking of the C–C bond, while Co and Fe are active sites for OH^−^-ion adsorption for further oxidation of ethanol decomposition fragments [83].

### 3.3. Ethylene Glycol Oxidation

Among the small organic molecules that can act as fuel for proton-exchange membrane fuel cells (PEMFCs), ethylene glycol is one of the most promising candidates. Ethylene glycol has low toxicity [28,90,161], low membrane penetration [25,85,90,108,161], high-energy-density [25,161] and relatively high reactivity in ambient temperatures [28,161], which are all valuable features for fuel in PEMFCs.

It is a clear, odorless and biodegradable liquid that is very soluble in water [90]. Pure, anhydrous EG is not aggressive towards most metals and plastics, which, combined with low vapor pressure and stability, simplifies its transportation and storage. The only requirement for tank materials for ethylene glycol is that they cannot contain phenolic resins since they are not resistant to EG [90]. Additionally, EG is safer to work with than methanol and ethanol because it has a higher boiling point and higher volumetric capacity (see Table 1). Furthermore, because of the larger size of a single EG molecule, the membrane crossover is much smaller than in the case of methanol, which enhances the process efficiency because of the weaker cathodic poisoning effect [25,85,90,108].

The process of ethylene glycol production has been known since 1859, but its industrial-scale production began during World War I when it was used during the production of explosive materials as a substitute for glycerol [90]. Currently, it is a very important chemical that is widely used, i.e., in the automobile industry as a cooling agent [25,90,162] and as a raw material for the production of polyester fibers [90]. Nowadays, it is produced via hydrolysis of ethylene oxide, with an annual production of 7 million tons (for 2012) [90]. Such large-scale production means that the supply chains are well developed, which simplifies the adaptation to the role of fuel for current sources [25,90,162].

Even though EG is a simple diol, its complete electrooxidation to carbon dioxide is quite complicated. Its full oxidation can be presented as follows [7,88,162,163]:

(CH_2_OH)_2_ + 2 H_2_O → 2CO_2_ + 10 H^+^ +10 e^−^ (23)

(CH_2_OH)_2_ + 10 OH^−^ → 2CO_2_ + 8 H_2_O +10 e^−^ (24)

(CH_2_OH)_2_ + 14 OH^−^ → 2CO_3_^2−^ + 10 H_2_O +12 e^−^ (25)

The complete oxidation of one molecule of ethylene glycol to carbon dioxide results in a gain of 10 electrons (in comparison, complete MOR results in a gain of 6 electrons, and EOR results in a gain of 12 electrons) [91].

The EGOR is a very sensitive reaction, and the process conditions, such as the temperature or acidity, can strongly influence its reaction rate.

An alkaline environment provides better conditions for EGOR from a kinetic point of view because such conditions enable the higher activity of EGOR molecules [164,165]. Furthermore, better results observed for EGOR in the base environment can be related to the easier electron transfer in the presence of hydroxide ions [166].

Free OH^−^ ions in the feed stream can be adsorbed on the surface of the electrodes [86]. Since hydroxide ions are present in the solution, they do not need to be produced in situ by water activation in the same amount as that in the acidic environment, which simplifies the overall process [86,91,162].

Hydroxide adsorbed on the surface of the electrocatalytic material enhances the oxidation of the poisonous CO intermediates and thus enhances the overall reaction efficiency [86,161,165]. The release of the adsorbed carbon oxide derivatives prevents the poisoning of the electrode and thus enhances the overall electrode reaction yield because more free active sites on the electrode surface area available for newly delivered fuel molecules. Therefore, the higher the amount of oxygen-containing species on the surface of the electrode is, the better the CO poisoning immunity of the electrode and, consequently, the better the system performance [161,165,167].

**Figure 6:**
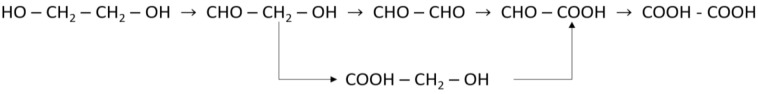
Mechanism of ethylene glycol oxidation to C_2_ intermediates based on [162].

The oxidation of ethylene glycol in alkali media can go through two paths: poisonous and non-poisonous [25,154], depending on the catalytic material used and the process conditions (i.e., pH and temperature) [154]. In the non-poisonous pathway, the main product of EGOR is oxalate, while for the poisonous one, products are formed during further oxidation of initially obtained formates [25,154]. Only some ethylene glycol molecules are fully oxidized, so the EGOR leads to a large number of intermediates containing two carbon atoms—C_2_ intermediates—including glycolic acid, glyoxal, glyoxylic acid, oxalic acid, glycolaldehyde and glyoxal [88,91,161,162,163,165] (Figure 6). The scheme of EG oxidation to C_2_ intermediates can be written as shown in Figure 6 [162].

The electrooxidation of ethylene glycol takes place in two steps. First, ethylene glycol molecules are adsorbed on the surface of the anode and dehydrogenated, which leads to the formation of C_2_ adsorbed products. This step is common for both the poisonous and nonpoisonous paths [154,161,162,163]. The second step of the EGOR is the oxidation of the formulated intermediates [25,154,161,162,163], and because they are less prone to oxidation than the original compound, this is the rate-determining step for the whole oxidation reaction [91,154,161,163]. At room temperature, the yield of ethylene glycol oxidation to CO_2_ does not exceed several percent, which makes it negligible [91].

The non-poisonous way leads to formation of oxalate (reaction (26)) [88]:

(CH_2_OH)_2_ + 10 OH^−^ → C_2_O_4_^2−^ + 8 H_2_O +8 e^−^ (26)

and a poisonous path leads to the formation of CO intermediates, such as glycolates (reaction (27)) and formate (reaction (28)), that are further oxidized into anode poisoning species [25,88,102,154,163].

The formation of glycolate is a four-electron reaction in which the C–C bond remains unbroken [163]:

(CH_2_OH)_2_ + 5 OH^−^ → CH_2_COO^−^ + 4 H_2_O + 4 e^−^ (27)

Another possible product, formate, is generated in a 6 electron reaction that involves the cleavage of the C–C bond [163]:

(CH_2_OH)_2_ + 8 OH^−^ → 2 HCOO^−^ + 6 H_2_O + 6 e^−^ (28)

The final nonpoisonous product is oxalate because of the absence of catalytic materials that are capable of oxidation with proper reaction kinetics [33,163]. Other products obtained during nonpoisonous EG oxidation include glycolate and glyoxalate, depending on the reaction pH, which is further oxidized to oxalate [88,154,163]. When EG is oxidized into oxalate species, 8 moles of electrons are obtained from one mole of EG (reaction (26). When we compare this to the maximum possible electron income from complete EG oxidation (reactions (24) and (25))—10 moles of electrons from one mole of EG—we obtain 80% current efficiency compared to full EG oxidation to carbon dioxide [88].

Fortunately, the partial oxidation of ethylene glycol can be advantageous. C_2_ intermediates, such as formates, glycolates or oxalates, are valuable organic compounds. Therefore, the lower energetic value of the partial oxidation process is compensated by the production of valuable chemicals that can be isolated from the product stream and further used in other industries for, for example, medicine, pesticides and organic synthesis [102,154].

Most of the established technologies for DAFCs are based on anodes made of platinum and its alloys [31,85,91,108,144,163,167,168,169,170,171]. Because of their popularity, their manufacturing process is well established, and the control of the metal ratio, alloy level and surface morphology is possible [115,162]. This well-established production technology makes platinum-based anodes perfect candidates for electrocatalytic materials and is the main reason for studying their catalytic activity towards the oxidation of various alcohols [115,162].

The electrooxidation of C2 alcohols, such as ethanol and ethylene glycol, with platinum-based anodes, can show low efficiency. This phenomenon is linked to the high sensitivity of Pt towards poisoning with carbon intermediates rather than to its inability to break the C–C bonds [91,154,161,163] and to favorable kinetics of partial oxidation to C2 intermediates compared to full oxidation to carbon dioxide [91,162].

To minimize the harmful influence of reaction products on platinum electrodes, anodic materials are doped with additives that significantly increase their immunity to poisoning. The doping of platinum anodes with other metals leads to modification of Pt geometric and electronic structures by Pt–M interactions, where M can be Sn [15,91,144,168], Pd [88], Au [167,172], Ru [85,108,163,173,174], Ni [163,169], Pb [161], Bi [88], Co [171], etc. and thus to changes in catalytic activities [161,162].

As mentioned, catalyze is a surface process. A bigger area of contact between the solution and the electrode enhances the reaction rate thanks to a higher amount of active sites accessible for alcohol particles and better conditions for their transport, enabling the diffusion towards the electrode [170]. To enhance its catalytic activity towards EGOR, different forms of anodic materials have been examined from solid electrodes, mesh [49] or aerogels [175] to immobilized nanoparticles, both mono- [144,174,176] and multimetallic ones [51,177,178]. Because of very promising results, most of the researchers have focused on developing catalytic nanoparticles. Thanks to their high surface-to-volume ratio, small catalytic particles show high reactivity related to increased mass and electron transport, which are usually the rate-limiting steps during alcohol electrooxidation [170]. Another advantage of this type of anodic material is its higher durability than that of a classic Pt/C electrode. The influence of support material on such properties cannot be omitted; Pt/r-GO/Nb_4_N_5_ is a material with high electrical conductivity and a large electroactive area because of the presence of carbon nanocompounds and niobium nitride. This material remains stable after cycling for 6000 s because of the protection of the r-GO layer [100].

One of the disadvantages of nanoscale catalytic materials is the fact that the performance of anodes based on nanoparticles can be easily influenced by changing the catalyst loading. The reaction potential remains constant because this change does not influence the reaction mechanism but rather accelerates the reaction by providing more active centers. As a consequence, greater catalyst loading often leads to an increase in the peak current, but in some cases, a greater loading of nanoparticles decreases the peak current because of the agglomeration of deposited nanoparticles, thus lowering the electroactive surface and consequently decreasing the reaction efficiency [88].

Similar to previous substances, ethylene glycol electrooxidation is sensitive to the geometric structure of the anodic material. The highest activity for EGOR is observed for platinum materials with a (111) plane structure, which is characterized by a low number of structural defects, which lowers the affinity of CO_ads_ to the electrode surface and eases their desorption. This enables high activity towards the oxidation of adsorbed carbon-based intermediates, thus improving the electrode immunity from catalyst poisoning [144,161,170].

The geometric structure is not the only property of the catalytic material that can strongly influence the EGOR. Other electrode material characteristics, such as the number of active reaction centers on the electrode surface and the electronic structure, which changes the rate of charge transfer, strongly influence the reaction rate. As mentioned, the carbon intermediates produced during EG oxidation can reduce the number of active centers available for EG particles by adsorption on the anode and thus decrease the reaction kinetics and efficiency [91,154,161,163].

Fortunately, the carbon intermediates can be desorbed from the electrode surface through their further oxidation to carbon dioxide during the second step of the EGOR. This reaction requires the presence of adsorbed hydroxide ions on the surface of the electrode. Oxophilic elements, such as Ru [85,162], Ir [108], Sn [15,91], Pd [88], Co [37] and Ni [108], enable water dissociation at potentials lower than that of pure platinum (0.35 V for Ru and 0.6 V for Pt); thus, this kind of catalytic material shows a bifunctional mechanism: alcohol is adsorbed on the platinum surfaces, and water is split on the surface of the ruthenium, which is presented below [85,108,161,162,179]:

(29) $$\mathrm{Pt}\;+\;({\mathrm{CH}}_{2}\mathrm{OH}{)}_{2}\;\overset{\;}{\to }\;\mathrm{Pt}({\mathrm{CH}}_{2}\mathrm{OH}{)}_{2}{\;}_{\left(\mathrm{ads}\right)}\;\overset{\;}{\to }\;2\;\mathrm{Pt}(:\mathrm{CHO})\;\overset{+\;4\;H_{2}O\;}{\to }\;2\;\mathrm{Pt}(\mathrm{HCOOH}){\;}_{\left(\mathrm{ads}\right)}\;+\;4\;H^{+}\;+\;4\;e^{-}$$

(30) $$\mathrm{Pt}(\mathrm{HCOOH}){\;}_{\left(\mathrm{ads}\right)}\;\underset{-\;\;H_{2}O}{\to }\;\mathrm{Pt}(\mathrm{CO}{)}_{2}\;{\;}_{\left(\mathrm{ads}\right)}\;$$

(31) $$\mathrm{Pt}\;+\;H_{2}O\;\underset{\;}{\to }\;\mathrm{Pt}(\mathrm{OH}{)}_{\mathrm{ads}}\;+\;H^{+}\;+\;e^{-}$$

(32) $$\mathrm{Ru}\;+\;H_{2}O\;\underset{\;}{\to }\;\mathrm{Ru}(\mathrm{OH}{)}_{\mathrm{ads}}\;+\;H^{+}\;+\;e^{-}$$

(33) $$\mathrm{Pt}(\mathrm{OH}{)}_{\mathrm{ads}}\;+\;\mathrm{Pt}(\mathrm{CO}{)}_{\mathrm{ads}}\;\underset{\;}{\to }\;\mathrm{Pt}\;+\;{\mathrm{CO}}_{2}\;+\;H^{+}\;+\;e^{-}\;$$

(34) $$\mathrm{Ru}(\mathrm{OH}{)}_{\mathrm{ads}}\;+\;\mathrm{Pt}(\mathrm{CO}{)}_{\mathrm{ads}}\;\underset{\;}{\to }\;\mathrm{Pt}\;+\;\mathrm{Ru}\;+\;{{\mathrm{CO}}_{2}}^{↑}\;+\;H^{+}\;+\;e^{-}\;$$

Although pure ruthenium shows no catalytic properties towards ethylene glycol oxidation at room temperature [162], it is still one of the most popular doping elements for Pt electrodes [108,162,163]. EGOR on the surface of a PtRu catalyst is controlled by the kinetics of adsorption of the fuel molecules on the anode and the desorption of the reaction product from its surface [173]. The composition of multimetallic electrodes is a very sophisticated process because catalyst composition strongly influences the properties of the final material. For example, in the case of a PtRu electrode, the activation energy for EGOR on a PtRu catalyst strongly depends on the Ru content; if too little ruthenium is doped into the electrode material, unfavorable adsorption kinetics take place. On the other hand, the addition of too much Ru strongly inhibits electrode activation [173,174]. Moreover, the addition of too much dopant, such as ruthenium or tin, may lead to the formation of separate metallic phases [168]. The maximum ruthenium content varies depending on the electrolysis environment. In acidic environments, with increasing Ru content, the onset potential for EGOR decreases. This phenomenon is probably caused by the optimum Pt:Ru ratio, which oscillates from approximately 15–20%, enabling the optimal ratio of three platinum adsorption sites to one ruthenium adjacent atom providing the optimal ratio of adsorbed hydroxide ions for oxidation of adsorbed ethylene glycol molecules [174]. In alkaline environments, the maximum ruthenium content is 50% because a further increase in its content leads to a decrease in the EGOR to CO_2_ reaction efficiency, which is related to ruthenium’s influence only on the main EGOR reaction but also on the parallel reactions [162].

The formation of ternary systems with catalytic activity towards EGOR is more complicated than in the case of ethanol or methanol oxidation. The addition of popular doping elements, such as nickel or palladium, has not influenced the activity of PtRu electrodes [49]. Different results are observed after the addition of tungsten into the Pt-Ru system. Usage of such ternary electrodes has led to higher peak current and lower reaction onset potential [49,162]. Such behavior has been explained by a bifunctional mechanism, in which tungsten is responsible for enhanced water dissociation [49,162]. It has not only naturally low activation energy towards water split reaction [49] but also thanks to its ability to form oxides with different oxidation states-WO_2_, W_2_O_5_ and WO_3_. Changing the oxidation states of tungsten can render active sites for water dissociative adsorption [49,162].

A similar situation takes place in the case of ruthenium and platinum oxides. Depending on the oxidation state of the metal, such molecules show different behaviors in electrolytic systems. They can either catalyze or inhibit the reaction. Ruthenium and platinum molecules containing metals in higher (Ru^IV^ and Pt^III^ and Pt^IV^) oxidation states slow the reaction. They are either very inactive towards EGOR or even prevent alcohol oxidation. Additionally, molecules containing these metals in lower oxidation states (0, I and II) are considered active species towards alcohol oxidation. For this reason, the stability of the electrode material is extremely important because its oxidation leads to lower activity and thus to an overall drop in the reaction efficiency [162].

Because platinum resources are limited, optimal usage of this strategic metal is necessary to lower the cost of anodic materials. One of the best strategies to obtain the best catalytic properties with the least platinum is the formation of core–shell structures. Because electrolytic oxidation is a surface process, the usage of platinum as a shell material enables favorable catalytic properties with the lowest possible platinum usage. In the core–shell particles, the core must consist of a material that is immune to a harsh fuel cell environment and cheaper than platinum so that the overall cost of the catalyst can be reduced [108]. Different metal combinations have been studied, such as Pt cores at Ru shells [85], Pt@Ru, PtRu@Ni or PtRu@IrNi [108], and PtRu or PtNi [163]. The composition of core–shell particles is even more prudent than that of classic electrodes. For example, preparation of Pt@Ru core–shell nanoparticles with almost no ruthenium present in the shell layer leads to structures that are less active than metallic PtRu catalysts because, without ruthenium, a bifunctional mechanism cannot take place, and thus, the current efficiency of the overall reaction decreases [85].

Core–shell particles are not the only catalytic nanomaterials that can be used for the electrooxidation of organic molecules, such as ethylene glycol. Furthermore, one-dimensional materials, such as nanowires [25] or nanofibers [15], can be applied. Their strong advantage is the fact that they are characterized by a very large electroactive surface (because of high volume to surface ratio), which allows better utilization of the catalyst and consequently lowers the amount of material necessary for electrooxidation, and high flexibility and stability, which allows the production of smaller, more portable devices [15,25].

Popular doping elements for platinum-based electrodes, other than ruthenium, include cobalt [171], palladium [88,154,162], bismuth [88,162], lead [162], tin [91,144,168], gold [25,58,162,167,180,181] and silver [31,51,182]. All of the mentioned metals change the anodic material properties in different ways. The doping of cobalt into platinum-based catalysts improves the conductivity, thanks to the electronic (ligand) effect, which improves the whole reaction’s efficiency [171]. The doping of palladium, as palladium shows catalytic properties towards the EGOR on its own, results in an extraordinarily active material with a large electroactive area because active centers for EGOR are present not only on platinum but also on palladium [162]. The addition of bismuth leads to the formation of C_2_ molecules as the main reaction products. This is probably linked to the dilution of active platinum centers and thus the weaker ability of the electrode to break the C–C bonds. The losses linked to doping the electrode material with bismuth significantly outweigh the benefits [88,162].

Tin, as an element that is less electronegative than platinum, gives its valence electrons to platinum, which results in the occurrence of an electronic (ligand) effect. This change in the Pt electronic structure strongly influences CO affinity to the anodic surface and thus improves the electrode immunity to poisoning [144]. Additionally, because of the high oxophilicity of tin [144], the water-splitting potential decreases as a consequence of the formation of tin oxides Sn(O_x_) [91,168] or hydroxides—Sn(OH)_2_ and Sn(OH)_4_ [144]—on the surface of the electrode material, which enhances the further oxidation of adsorbed carbon intermediates and consequently improves the anodic efficiency and immunity to poisoning even more [91,144,168]. Another advantage of using PtSn as an anodic material is that it shows catalytic properties towards EGOR and ethanol and glycerol oxidation. This versatility would be very convenient for potential industrial applications because it would allow fuel changes depending on the availability or price of these fuels [15].

The EGOR mechanism on the surface of the PtSn anode involves the formation of *COCH_2_OH as an intermediate before the breaking of the C–C bond [91]. The presence of tin, which shows oxophilic properties, allows double site adsorption of this molecule, which enhances the selectivity towards carbon dioxide formation. This C–C split probably takes place by the adsorption of *COCH_2_OH through the carbonyl group on the Pt atom and by the oxygen connected to the beta carbon atom connecting to the tin atom [91]. This mechanism can be proven by analyzing the effect of alkali treatment on the PtSn catalyst. After such an operation, the selectivity of the catalyst towards CO_2_ formation decreases. This can be caused by the lack of free Sn adsorption sites, which are all occupied by hydroxide ions. Without the adsorption of hydroxy acetyl on tin, the adsorption on the Pt sites weakens, which makes the desorption and oxidative removal of carbon oxide intermediates more favorable. Consequently, the formation of mainly C_2_ intermediates lowers the electrode selectivity towards CO_2_ [91].

Another doping material for platinum electrodes is gold [58,162,167,180]. As a metal that shows good electrical conductivity and a strong affinity to oxygen, it has characteristics favorable for a doping material [167]. Doping platinum electrodes with gold gives the final material higher activity thanks to the synergistic effect [180] and stability in alkaline environments, which is crucial for the EGOR and enhances the kinetics under these conditions [162,167].

**Figure 7:**
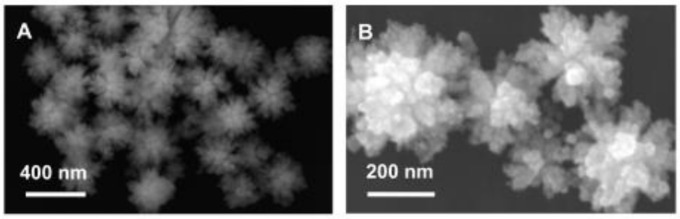
Low- (**A**) and high-magnification (**B**) SEM images of dendrite-like PtAg nanocrystals, reprinted with permission of Elsevier [31].

Silver, as a metal with the highest electrical conductivity and high oxophilicity, also has been proposed as a doping agent for platinum catalysts for EGOR. This has led to catalytic materials with higher activity and stability than pure platinum thanks to enhanced conductivity [31,182]. An example of PtAg nanostructure is presented in Figure 7.

The addition of two metallic, oxophilic elements lowers the water activation energy even more and thus lowers the EGOR onset potential [162]. For this reason, ternary systems, such as PtPdBi [88,154], PtRuNi, PtRuW and PtRuPd [162], have been developed. Such electrodes are more resistant to poisoning with carbon intermediates because of the optimal distribution of EG and OH species due to the presence of palladium and water activation on the bismuth atoms. When nickel is added to the PtSn system, this doping enhances the catalytic activity of the anode because of the changes in Pt electronic structure and NiO formation on the electrode surface, which results in a combination of the electronic effect and the bifunctional mechanism [162]. Compared to platinum catalysts, such electrodes lead to enhanced oxalate formation, which is linked to a lower surface tendency for catalyst poisoning with CO intermediates [88,154,162].

The second group of popular electrocatalytic materials for EGOR is based on palladium. Even though this metal is more abundant than platinum [22,36,39,183], its price is higher [22], but because of its unique catalytic properties and higher stability, this disadvantage can be balanced by the higher efficiency of the oxidation processes [22]. During the oxidation of polyalcohols on the surface of palladium-based materials, higher peak current densities than in the case of platinum-based electrodes are observed, and the stability of such materials in alkaline media, which provide favorable conditions for such reactions, is remarkable [22,36,63,183].

Similar to other catalytic materials, the activity of palladium-based electrodes can be enhanced by doping with other elements that can either modify the surface process mechanism [63], such as the bifunctional mechanism [22,39] or change the electronic structure of the main metal [36,148,183,184], thus improving its catalytic performance. Other modifications can be carried out by using highly conductive support materials, which can improve the electron transfer between the electrode and adsorbed molecules [36,183,185], or by preparing catalytic materials with high numbers of surface defects, which can improve the charge and mass transfer and decrease the energetic barriers in anodic materials [22,184].

Different doping metals change the reaction rate through different mechanisms. Dopants, such as bismuth [39,154] or nickel [22,186,187] in palladium result in a bifunctional mechanism that involves the oxidation of alcohol on the palladium atoms and water activation taking place on the surface of the doping elements. This provides the hydroxide ions necessary for complete alcohol oxidation. Different mechanisms of electrocatalytic activity enhancement are synergistic effects. These effects take place when doping elements, such as iridium [188] or gold [36,57,189,190], induce an upshift in the palladium d-band center and thus result in stronger adsorption of hydroxide ions, which are crucial for complete alcohol oxidation [36,188,190]. This mechanism is similar to the electronic effect that takes place after doping with elements, such as copper [32,184], bismuth [39,154], iron [33,63,114,154,186] or ruthenium [24,39]. Instead of increasing the adsorption of hydroxide ions, an electronic effect takes place; in the breaking of inter-carbon bonds, the charge transfer and desorption of CO-based intermediates are improved [24,39,184].

Much attention has been focused on Au–Pd catalysts because of their excellent catalytic properties towards the hydrogenation of acetylene, the synthesis of acetate and the oxidation of alcohols. The presence of gold lowers the cost of the final catalytic material and its sensitivity towards poisoning with carbon oxide intermediates. This improvement is related to the synergic effect that takes place between gold and palladium in such materials. Gold induces an upshift of the palladium d-band center, which results in a stronger affinity for hydroxide ions. This effect enhances the adsorption of hydroxide ions and improves the kinetics of the EGOR [36,190].

Because of its relatively low price and very interesting properties, electrodes for the electrooxidation of EG based on gold, as the main metallic ingredient, have also been developed. This element shows catalytic properties not only towards the EGOR but also towards providing the hydroxide ions that are necessary for high-performance of the catalytic material and its immunity to poisoning [25,28,57,86,175,176,191]

As with any of the other already-mentioned materials, gold-based catalysts show higher activity when their electroactive surfaces are increased. The easiest way to significantly increase the active area of the electrode is to increase its surface-to-volume ratio using electrode materials in the form of nanocompounds. Due to the sophisticated methods of their synthesis, gold-based nanomaterials are easy to shape and form, which allows simple alteration of ECSA. High electroactivity enhances the reactivity of the electrode material and improves the surface atom utilization, which allows the use of smaller doses of the catalyst for the same result, lowering the cost of the overall process [25,30,86,176,181,191].

Despite its catalytic properties, gold is rarely used as a catalytic material on its own because of its poisoning with reaction product and poor stability, both leading to a decrease of the reaction active centers [25,86,175]. Currently, gold catalysts are usually used with doping agents that enhance their performance [25] or with supporting materials that alter the catalyst properties [86].

One of the doping agents that can be used to improve the gold catalyst performance is silver. AuAg alloys show a bifunctional mechanism: on Au sites, alcohol molecules are adsorbed and oxidized, while on the surface of Ag, oxygenated species are promoted [25].

Metal oxides, such as CeO_2_, Fe_2_O_3_ and RuO_2_, used as embedding for gold catalysts, can also enhance the catalytic performance of gold nanoparticle catalysts [86]. Their highly oxophilic character provides conditions for the occurrence of a bifunctional mechanism that enhances the catalyst performance by improving the adsorption of hydroxide ions on the catalyst surface [86]. The use of metal oxides as support materials for golden nanoparticles simplifies the preparation of the catalytic system, which requires the formation of only one nanocompound while maintaining the benefits of these oxides as doping agents. However, this solution, as always, has flaws—too high an amount of iron and ruthenium oxide leads to a decrease in the electroactive catalyst, which is probably related to the formation of clusters from gold nanoparticles [86]. In addition, catalyst stability can decrease as a result of the presence of ruthenium oxide because it can over-provide the catalyst with oxygen species, which leads to surface poisoning with Au_2_O_3_–gold(III) oxide, which shows no catalytic properties towards EGOR and blocks bulk ethylene glycol molecules from adsorption on the electrode surface [86].

Additionally, the combination of platinum and gold has shown excellent results towards EG electrooxidation. Both of these metals have catalytic properties towards EGOR, and gold has an additional ability to prevent adsorption of the reaction intermediate products on the platinum surface, which protects the active centers of the catalyst from poisoning [167,172].

Different kinds of palladium-based nanoparticles have been developed, from simple palladium nanoparticles [183] and PdNi nanocubes [22] to nanoflowers made of palladium and silver [192], to complicated ternary core–shell systems, such as FeCo@Fe@Pd particles [33]. Despite their differences related to different compositions, they are all characterized by high electroactive surface related to high volume to surface ratio. Nanoporous catalytic materials have also been examined as catalytic materials for the oxidation of small organic molecules. They can be relatively easily obtained by selective dissolution of the active phase from the alloy-dealloying process. For example, TiCu amorphous alloys are de-alloyed in nitric acid solution [166]. This material was additionally treated at high-temperature. The final material shows significant activity towards EGOR in both acidic and alkaline media. Untreated TiCu alloy shows no catalytic activity in alkaline media and low activity in alkaline solution. Heat-treated nanoporous materials show better results in terms of both catalytic activity and stability, which is probably related to less homogenous and larger pore sizes. Larger pores enable the diffusion process, which enhances the overall reaction rate [166].

### 3.4. Propanols

The term propanols refer to two isomers: 1-propanol and 2-propanol, also called isopropanol. Both are clear liquids with characteristic smells and low vapor pressure. This last feature has promoted their use as solvents on an industrial scale. Other applications of propanols include anti-freezing agents, biocidal agents and substrates in organic syntheses, such as the production of esters or amines.

The larger production scale of isopropanol is linked to its larger industrial significance [92]. Additionally, isopropanol can be obtained from biomass materials, making it more environmentally friendly than the linear isomer [59,193]. Both propanol isomers on an industrial scale are produced by hydrogenation—isopropanol is a result of the hydrogenation of acetone (reaction (36)), and propanol is obtained by the hydrogenation of propanal (reaction (37)) [92]:

CH_3_C(O)CH_3_ + H_2_ → CH_3_CH(OH)CH_3_ (35)

CH_3_CH_2_CHO + H_2_ → CH_3_CH_2_CH_2_OH (36)

Saturated C_3_ alcohols, especially isopropanol, which is the smallest secondary alcohol, have been of great interest as potential fuels for fuel cells. They are less toxic than methanol, and the direct alcohol fuel cells that use them for electricity production perform better than DMFCs because of the much lower crossover current, which is limited due to the size of C_3_ alcohol molecules, which are larger than methanol [59,193,194,195,196,197].

Electrooxidation of propanol is possible in both acidic and alkaline media on palladium- or platinum-based catalysts. Platinum shows greater activity towards propanol oxidation, while palladium gives better results as an anodic material for isopropanol oxidation [130]. The main product of propanol oxidation is propanal, and the isopropanol product stream consists of almost only acetone. The presence of these high molecular weight products is related to the high stability of inter-carbon bonds, which are very difficult to break on the surface of the electrode [135,161,195,197,198,199]. The oxidation of both propanol isomers is possible with the use of a PdAg catalyst. Even though both isopropanol and propanol undergo oxidation on their surface, a higher current density is observed for primary alcohols [178]. This difference is probably linked to the conformation of these alcohols. The presence of silver in palladium-based catalysts results in weaker adsorption strength holding oxidation intermediates on the surface of the electrode, which results in higher immunity to CO intermediate poisoning in the final electrocatalytic material [178].

The oxidation of aliphatic alcohols results mainly in the corresponding aldehydes and CO_2_ [135].

The overall 1-propanol electrooxidation reaction can be expressed as follows [135]:

CH_3_CH_2_CH_2_OH → CH_3_CH_2_CHO + 2 H^+^ + 2 e^−^ (37)

CH_3_CH_2_CH_2_OH + 5 H_2_O → 3 CO_2_ + 18 H^+^ + 18 e^−^ (38)

As visible in reaction (37), the usage of a feed stream that consists only of propanol results in propanal as a product and a small anodic current. For full propanol oxidation towards carbon dioxide (reaction (38)), the presence of water in the feed stream is necessary. The amount of released electrons is nine times higher than that in the case of pure propanol oxidation. This difference is caused by the presence of oxygen atoms in water molecules, which is necessary for carbon dioxide formation [135].

Isopropanol can be electrochemically oxidized in both acidic and alkaline media [195,198]. Its electrooxidation in acidic media results in obtaining higher current densities than MOR in the same conditions [200]. For alkaline solutions, observed peak current densities are even higher because the higher pH value of the electrolyte enhances the oxygen reduction reaction that takes place on the cathodic site of the fuel cell [198,201].

The overall 2-propanol electrooxidation reaction, despite the reaction environment, can be expressed as [135,161,195,198,202]:

CH_3_CH(OH)CH_3_ → CH_3_C(O)CH_3_ + 2 H^+^ + 2 e^−^ (39)

CH_3_C(O)CH_3_ + 16 OH^−^ → 3 CO_2_ + 11 H_2_O + 16 e^−^ (40)

As shown in reactions (39) and (40), acetone formation strongly prevails during 2-propanol electrooxidation. In the products stream, only a little amount of CO_2_ is detected [161,198]. The absence of CO and its intermediates is one of the greatest advantages of isopropanol as a fuel because it lowers the chances of occurring of the phenomenon of self-poisoning of the system with CO intermediates and allows power generation without carbon dioxide emission [197,199]. Additionally, isopropanol’s oxidation to acetone takes place in a lower potential region than its complete oxidation to CO intermediates and further to carbon dioxide, which makes it more efficient [198,199].

Oxidation to acetone, instead of full oxidation to carbon dioxide, is one of the greatest advantages of direct isopropanol fuel cells. Because of the lack of CO_2_ emissions, this kind of fuel cell is carbon neutral and thus is even more environmentally friendly than other fuel cell technologies [197,199]. Additionally, because of this reaction, the isopropanol and acetone system can act as a liquid hydrogen carrier—a pair of hydrogen-rich (isopropanol) and hydrogen lean (acetone) molecules that can be used as hydrogen sources with repeated catalytic hydrogenation and dehydrogenation cycles. In the fuel cell, the role of the catalyst is played by the electrocatalytic materials, from which the electrodes are made. Such a solution can be very convenient because hydrogen as a fuel has many interesting features, such as high gravimetric energy storage, and enables fully de-fossilized energy production [197,199]. Because classical approaches to hydrogen storage, such as compression or cooling, do not seem to be effective, other methods of hydrogen storage must be developed. Liquid hydrogen carriers combined with fuel cells can be an elegant solution to this problem—in one device and on one electrocatalytic material, both reactions (protonation and deprotonation) can take place, which allows better usage of space and lower investment costs. Additionally, using electrocatalytic materials for the deprotonation of organic molecules in fuel cells results in protons instead of hydrogen molecules, which increases the system’s safety [197,199].

The cell reactions using this system can be described as [197]:

(A): CH_3_CH(OH)CH_3_ → CH_3_COCH_3_ + 2H^+^ + 2e^−^ (41)

(C) 2H^+^ + ½ O_2_ + 2e^−^ → H_2_O (42)

The summary reaction is as follows:

H_3_CH(OH)CH_3_ + ½ O_2_ → CH_3_COCH_3_ + H_2_O (43)

The theoretical potential of such a cell (1.1 V) is 13 mV lower than that for the classic hydrogen fuel cell (1.113 V) but is higher than the potentials obtained for other direct alcohol fuel cells fed methanol or ethanol [197].

The efficiency of isopropanol oxidation on platinum- and platinum-based catalysts in alkaline media is very low, mainly because of its lack of stability under reaction conditions, high sensitivity to poisoning and lack of ability to break the inter-carbon bonds in isopropanol molecules, which is why, for 2–propanol oxidation, mainly palladium-based catalysts are used [130,161,194,198,203].

Monometallic electrodes show worse results in terms of electrocatalytic effects towards alcohol oxidation and isopropanol oxidation on palladium, which is not an exception to this rule. Even though palladium shows good catalytic properties, such as low onset potential and high current density, acetone, which is the main product of this reaction, can strongly adsorb on the surface of the electrode and thus prevent the adsorption of fresh portions of isopropanol from the bulk solution, which leads to a decrease in the system efficiency [135,194,198,202,203]. Just like in the cases of other catalytic materials, the palladium activity can be improved by doping with other elements that show catalytic properties towards isopropanol oxidation and strong immunity to poisoning, like, for example, nickel [198] or iron [59].

**Figure 8:**
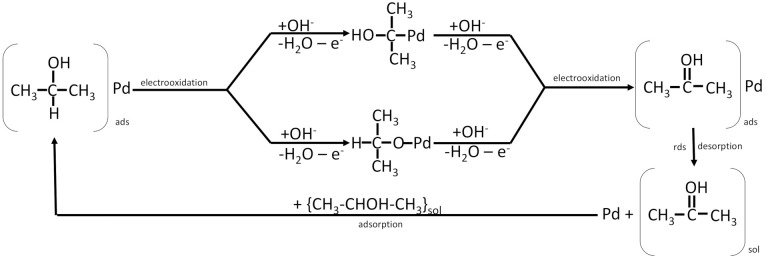
Reaction mechanism for isopropanol oxidation on Pd/Ni electrode reprinted with permission of Elsevier [198].

The mechanism of isopropanol oxidation of PdNi catalyst is presented below, in Figure 8.

The addition of iron into palladium-based catalysts also enhances their catalytic and anti-poisoning properties for regimes focused on obtaining CO_2_ as the main product. Fe can enhance the desorption of carbon oxide-based intermediates from the electrode’s surface, thanks to the electronic (ligand) effect, and therefore, provide higher stability and longer activity to the catalyst [59].

Some researchers have shown different approaches, and instead of palladium-based electrodes, they have developed Pt-based electrodes with other metal additives that significantly change the poisoning sensitivity and ability to break inter-carbon bonds. Doping with metals, such as lead [161], palladium [130], nickel [198], ruthenium [161,197,199] or gold [161,194,196,201,202] leads to occurrence of bifunctional mechanism.

The addition of electron donor molecules, such as Ni_2_P, to platinum catalysts, provides stability for the resulting material thanks to the occurrence of the electronic (ligand) effect. Ni_2_P provides electrons that stabilize the platinum atoms and lower the adsorption energy for isopropanol. This results in a final material with higher activity and stability (than the pure Pt/C catalyst). Nickel phosphide also shows the ability to prevent the agglomeration of platinum particles, leading to better utilization of the noble metal used and a higher active area in the final electrode material [193].

**Figure 9:**
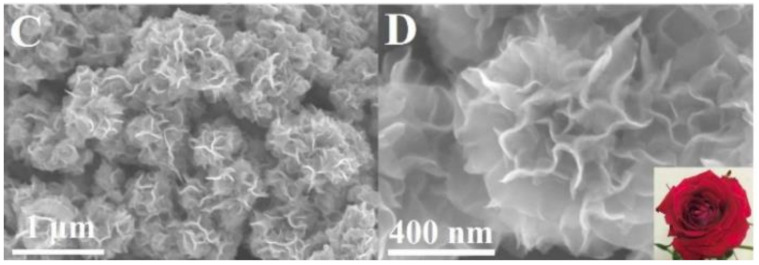
Low- (**C**) and high-magnification (**D**) SEM images of rhodium nanoroses, reprinted with permission of Elsevier [204].

Additionally, other non-noble metal-based materials have been investigated, such as rhodium, which shows catalytic properties towards isopropanol electrooxidation on its own, especially when the obtained material is on the nanoscale, like rhodium nanoroses presented in Figure 9, and can be characterized by a high ECSA [204], or titanium dioxide, which shows photocatalytic properties [205]. After doping TiO_2_ with transition metals, such as copper, and the final system shows electrocatalytic and photocatalytic properties towards isopropanol oxidation [205].

## 4. Comparisons of Alcohols Oxidation

Comparison of simple alcohols containing only one hydroxide group is presented in Table 2 [135]:

The electrochemical activity of these alcohols to oxidize towards CO_2_ decreases in order: methanol, ethanol 1-propanol, 2-propanol.

The largest gap is visible between methanol and ethanol, which probably reflects that the formation of CO_2_ from alcohols containing 2 or more carbon atoms requires the cleavage of at least one C–C bond. This thesis is strongly supported by the similar CO_2_ amounts obtained as a result of ethanol and 1-propanol oxidation, which both require breaking of one C–C bond, and the lack of CO_2_ present in the product stream for 2-propanol oxidation, which requires the breaking of 2 C–C bonds.

Additionally, under prototype alcohol fuel cell conditions, the formation of the corresponding aldehydes dominates ethanol and propanol oxidation. This is compatible with the view that aldehydes are formed via weakly adsorbed intermediates and that the strongly adsorbed intermediates are the precursors of carbon dioxide. In the case of methanol, the strongly adsorbed species constitute almost only CO and related species, whose oxidation probably proceeds more rapidly than the strongly adsorbed intermediates of ethanol and 1-propanol. This hypothesis explains the similarity between the oxidation of propanol and ethanol and why the water-to-methanol ratio in the feed stream strongly affects methanol oxidation. During C_2_ and C_3_, primarily alcohol C–C cleavage and the subsequent oxidation to CO_2_ are the most important factors influencing product distribution, while for methanol, where no C–C bonds must be broken, the amount of available water becomes the most important factor for product distribution.

## 5. Conclusions

The development of new electricity production methods is one of the greatest challenges humanity will face in the 21st century. Fuel cells are a concept that can be a solution to this problem. The use of low molecular weight alcohols as a fuel for such devices can be very convenient because they show not only high energetic densities but are also easy to store and transport. Their production streams are also well developed.

The best results so far have been observed for nanoscale materials because of their low resistivity and well-developed surfaces related to a high surface-to-volume ratio, which leads to high electrochemically active surfaces. This feature is crucial for developing smaller, more portable devices that will have greater chances for commercialization. The most important conclusions for each of described alcohols are shown below:

1. Methanol is considered the most likely fuel for industrial-scale fuel cells because it is the smallest alcohol, and its oxidation leads to carbon dioxide and water;
  - It can be oxidized in both acidic and alkaline environments on platinum-based electrodes, mainly with the addition of ruthenium;
  - The main problem with this kind of electrode material is that it can easily be poisoned with intermediate products and low reaction kinetics. If we also consider platinum shortages and their consequent high prices, it becomes clear that other electrocatalytic materials must be developed;
  - Nickel- and cobalt-based materials have the greatest chance of replacing platinum-based electrodes because of their low price, high activity and immunity to poisoning with carbon oxide intermediates;
  - Problems exist during methanol electrooxidation in addition to those associated with the electrode materials. Because of this particle’s small size, methanol can crossover the membrane, separating the anodic and cathodic parts of the fuel cell, which results in lower efficiency of the whole system.
2. Ethanol, which has only one more carbon atom than methanol, is an obvious candidate for this role;
  - Ethanol can also be oxidized in both acidic and alkaline environments, mainly on platinum catalysts, but these catalysts are doped with tin;
  - The oxidation of ethanol is more complicated than that of methanol because it requires the breaking of strong, inter-carbon bonds—the same feature that gives ethanol its stability and makes it an interesting fuel is the main cause of problems during its oxidation. Additionally, in this case, catalyst poisoning can deactivate the electrodes;
  - Other materials have been developed—palladium-based electrodes doped with oxophilic elements, such as copper, silver or nickel, have yielded very interesting results;
3. Because C–C bonds are so hard to break for larger alcohol molecules—such as ethylene glycol (the smallest diol) and isopropanol (the smallest secondary alcohols)—different approaches have been taken. The main goal is not their full oxidation to carbon dioxide but to valuable intermediates;
  - The products of ethylene glycol oxidation, such as glycolates and formates, can be marketed as substrates for other processes;
  - Isopropanol oxidation, which leads to the formation of acetone, can be coupled with its hydrogenation and thus can play the role of a liquid hydrogen carrier;
  - For both alcohols mentioned in point 3, electricity production can take place without carbon dioxide emissions, and thus, it can be more environmentally friendly than previously described systems. Such reactions require selective catalysts that guarantee that only the desired products are obtained;
  - For both, this effect is observed for palladium-based electrodes doped with oxophilic elements, such as gold, copper or nickel.

## Figures and Tables

**Table 1:** Physical properties of mentioned alcohols.

Alcohol	Density (kg m^−3^) *	Boiling Point (°C) **	Energetic Density (MJ L^−1^)	Heat of Combustion (MJ kg^−1^)	Theoretical Energetic Density (kWh kg^−1^)	E° Cell (V)
Methanol	786.68 [2]	64.70 [84,85]	17.85 based on [2]	22.69 [2]	6.1 [86,87,88]	1.213 [87]
Ethanol	789.30 [84]	78.32 [84]	23.49 based on [84,89]	29.76 based on [89]	8.00 [23,86,87]	1.145 [87]
Ethylene glycol	1113.50 [90]	197.60 [85,90]	21.23 based on [90]	19.07 [90]	5.2 [23,86,88,91]	1.22 [91] 1.029 [87]
Propanol	803.60 [92]	97.22 [92]	27.00 based on [92]	33.60 [92]	5.58 [87]	1.067 [87]
Gasoline					10–11 [87]	

* At 20 °C, ** for 1013.25 hPa.

**Table 2:** Comparison of the properties of simple alcohols.

Property	Methanol	Ethanol	Propanol	Isopropanol
Oxidation OCP vs. RHE, V	0.1	0.11	0.12	0.18
% CO_2_ in product stream (for stoichiometric water content)	87	27.4	19.5	0

## Data Availability

Data is contained within the article.

## Abbreviations

**List of used symbols**	
CNC	Carbon nanocages
CNT	Carbon nanotubes
CO_ads_	Adsorbed carbon oxide intermediates
DAFC	Direct alcohol fuel cell
DMFC	Direct methanol fuel cell
ECSA	Electrochemically active surface
EGr	Exfoliated graphite
EG	Ethylene glycol
EGOR	Ethylene glycol oxidation reaction
ESA	Electrode surface area
EOR	Ethanol oxidation reaction
GC	Glassy carbon
GNS	Graphene nanosheets
MOR	Methanol oxidation reaction
MWCNT	Multi-walled carbon nanotubes
OH_ads_	Adsorbed hydroxide ions
OCP	open-circuit potential
PEM	Proton-exchange membrane
PEMFC	Proton-exchange membrane fuel cell
rGO	Reduced graphene oxide
RHE	Reversible hydrogen electrode
SEM	Scanning electron microscope
TEM	Transmission electron microscope
